# General practitioners’ attitudes towards liquid biopsy as a diagnostic test for advanced lung cancer

**DOI:** 10.1016/j.jlb.2025.100330

**Published:** 2025-10-13

**Authors:** Danyon Lo, Jenny Wong, Rawiri Keenan, James Fingleton, Annie N.M. Wong

**Affiliations:** a University of Otago, Department of Medicine, PO Box 7343, Wellington, 6242, New Zealand; b University of Waikato, Division of Health, Hillcrest Road, Hamilton, 3240, New Zealand

**Keywords:** Circulating tumour DNA, Liquid biopsy, Molecular test, General practice, Lung cancer

## Abstract

**Background:**

In New Zealand (NZ), lung cancer disproportionately affects our indigenous Māori population, with both incidence and mortality being three times higher than NZ Europeans. Incidence of endothelial growth factor receptor (*EGFR*) mutated lung cancer is two to three times higher in Māori, Pacific and Asian people compared to NZ Europeans. Outcomes could potentially be improved if testing was augmented by biomarker testing of circulating tumour deoxyribonucleic acid (ctDNA) at the outset of diagnosis. Testing as a liquid biopsy could be more accessible, faster, and lower cost compared to tissue biopsy.

We aimed to evaluate NZ General Practitioners' (GPs’) attitudes towards liquid biopsy.

**Methods:**

A survey was conducted of NZ GPs and was distributed through online channels between November 2023 to January 2024.

**Results:**

Seventy GPs responded and challenges reported with the diagnostic process of lung cancer were: limited GP appointments, poor access to imaging, and limited access to secondary care. 53/58 (91 %) of GPs were unaware of liquid biopsy. 46/58 (79 %) were initially not comfortable with pre-test genetic counselling associated for this. Some GPs highlighted the potential of liquid biopsy to complement existing procedures particularly in rural areas although concerns were expressed regarding culturally appropriate pathways. Provided adequate training and funding for liquid biopsy, 42/58 (72 %) of GPs stated they would be comfortable requesting liquid biopsy and the associated counselling.

**Conclusion:**

Most GPs were not familiar with liquid biopsy but 72 % supported these tests to be incorporated into current pathways. Liquid biopsy could potentially reduce diagnostic delays and inequities in lung cancer.

## Highlights

• 70 general practitioners were surveyed on using liquid biopsy to diagnose lung cancer.
• Only 12 % & 9 % of those surveyed were aware of molecular testing and liquid biopsy.
• 39 % of those surveyed were familiar with molecularly targeted treatments.
• 72 % of those surveyed would use liquid biopsy to test circulating tumor DNA.
• Education and funding are needed to apply liquid biopsy in the community setting.

## 1 Background

Lung cancer is a global health priority given it is the leading cause of cancer-related death. This is particularly true for New Zealand (NZ), where lung cancer disproportionately impacts the indigenous Māori population, with the incidence and mortality being three to four times higher in Māori [1]. Māori and Pacific patients are more likely to present with more advanced disease and are particularly disadvantaged with poor access to healthcare [2,3].

Improving the diagnostic process of lung cancer is the first step to accessing timely and effective treatment. In non-small cell, non-squamous lung cancer, endothelial growth factor receptor (*EGFR*) mutations are present in 20 % and anaplastic lymphoma kinase (*ALK*) rearrangements are present in 3–5 % of cases. Although, the rates vary by ethnicity, with *EGFR* being present in half of cases in an Asian cohort [4]. A recent NZ study found that the population risk of *EGFR* mutated lung cancer was significantly higher in Māori, Pacific and Asian patients compared to NZ Europeans [5]. Thus, improvements in early diagnosis and molecular testing are important strategies to address the ethnic disparity in lung cancer outcomes.

Pathological diagnosis and staging can take months after initial symptoms with frequent initial diagnosis occurring in an emergency setting [3]. In NZ, 40 % of lung cancers are diagnosed after an acute presentation to secondary care [6]. Of these patients, 59 % had seen their general practitioner (GP) within the preceding six months, reflecting an opportunity for earlier diagnosis [6,7]. Patient perspectives on barriers encompassed poor relationships with GPs, long wait times for appointments, investigations, referrals, and accumulating costs with appointments [8].

Ideally, the diagnostic pathway should comprise of a GP referral to secondary care and involve specialist input within 14 days [2]. Patients presenting to the GP with symptoms or signs suspicious of lung cancer will have investigations such as blood tests, chest X-ray and sputum cytology. Regional differences exist and some have access to same day X-ray imaging or access to a direct chest computed tomography (CT) without requiring advice from a respiratory physician or radiologist through a ‘fast track’ pathway.

The need for complex imaging and histological diagnosis can contribute to a source of diagnostic delay. Scenarios which cause this delay include: patient co-morbidities or extensive illness preventing a patient from undergoing procedures, insufficient or inadequate tissue biopsy samples which may require a repeat procedure, or limitations around staff and equipment [8]. Complications such as pneumothoraxes, bleeding and intubation can also arise from invasive diagnostic procedures and may result in a delayed or absent histological diagnosis.

Pathological diagnosis and subsequent molecular testing are crucial in the decision-making process for treatment [6]. Approximately 15–20 % of patients ultimately never acquire a pathological diagnosis, therefore miss out on optimal and personalised treatment [9]. Proposed strategies to reduce delays to treatment have included concurrent testing of both tissue and plasma to identify actionable oncogenes [10,11]. This signifies potential application across several clinical settings: At the time of suspicion of lung cancer in the community setting, presentation at emergency department, or where tissue sampling is not feasible. In NZ, there are opportunities to involve local Māori health care providers or provision of testing in Māori marae (traditional meeting halls)-based clinics to improve uptake [12].

There are now three comparative studies that have highlighted the high concordance of circulating tumour deoxyribonucleic acid (ctDNA) with tissue-based testing for lung cancer [[13], [14], [15]]. Mutations detected include: *EGFR* p.T790M*,* p.G719X*,* and p.L858R mutations*,* as well as *EGFR* exon 20 insertions, exon 19 deletions, exon 18 deletions*,* and *ALK* rearrangements. These studies also show a faster turnaround time and additional detection of actionable mutations by approximately 20 %. More recently the Canadian single-centre ACCELERATE study evaluated the use of concurrent ctDNA in patients with suspected lung cancer, again reporting the median time to treatment had reduced from 62 days to 39 days [16].

Liquid biopsy involves the analysis of ctDNA from a blood sample, providing a non-invasive method to detect genomic alterations associated with lung cancer. From the peripheral blood sample, ctDNA is extracted and analysed using either targeted polymerase chain reaction assays or broader next-generation sequencing panels. These platforms can detect key mutations, such as *EGFR* and *ALK*, with high specificity and high sensitivity in advanced lung cancer [[13], [14], [15], [16]].

Integration into practice comes with clinical limitations. Liquid biopsy is not a replacement for histological diagnosis, and it cannot provide information on tumour morphology or stage. Sensitivity may be reduced in early-stage disease or in patients with low ctDNA shedding. Furthermore, while ctDNA testing is highly effective at detecting mutations such as *EGFR* and *ALK*, it does not detect all mutation types as reliably as tissue-based methods. In NZ, there is currently no standardised platform for biomarker testing for lung cancer and the technology required for next generation sequencing for cancer is limited to research settings [12,17].

Given that patients with suspected lung cancer are likely to encounter GPs in initial stages of diagnostic pathway, there is a window of opportunity to utilise plasma ctDNA to reduce time to treatment. In this study, we sought to evaluate the understanding and attitudes of NZ GPs on the role of liquid biopsy for lung cancer.

## 2 Methods

A National survey was distributed to NZ GPs through NZ Doctor Publication, Royal College of General Practitioners bulletin, Best Practice Advocacy Centre bulletin, GP newsletters, and social media (full survey in supplementary data). Participants were provided with a direct link to the online survey which allowed them to access the survey questions. The questions encompassed GP practice demographics, number of patients with lung cancer under the practitioner's care, data on current diagnostic and treatment of lung cancer in their region, and perceptions on the understanding and use of liquid biopsy. The basic concepts around using liquid biopsy and molecular testing for lung cancer was explained briefly within the survey.

The survey was open to circulation between November 2023 to January 2024 and GPs completed this via research electronic data capture (REDCap). GPs voluntarily participated in this study with provision of an information sheet that outlined the purposes of the study. Consent was obtained through GPs commencing the survey. No incentivisation was given to participate in the survey although a copy of a participant's answers was offered immediately upon completion. Descriptive analysis of the data was performed and reported in aggregate.

Ethics approval was obtained via the University of Otago Human Ethics Committee (Reference number: D23/265)

## 3 Results

A total of 70 survey responses were returned, 59 were complete and 11 were partially complete with records being excluded from analysis for the questions where no response was provided.

The respondents covered most regions of NZ: 14 from Auckland (21 %), 13 from Waikato (19 %), 12 from Wellington (18 %), 5 from Canterbury (7.5 %), 5 from Northland (7.5 %), 4 from Otago (6 %), 4 from Marlborough (6 %), 3 from Bay of Plenty (4.5 %), 3 from Hawkes Bay (4.5 %), 2 from Taranaki (3 %), 1 from Manawatū-Whanganui (1.5 %), and 1 from Southland (1.5 %) regions. Surveyed GPs practiced mostly in urban areas (78 %) with the rest in rural areas (22 %). Regarding socioeconomic coverage, 25 % of GPs worked at government subsidised “very low-cost access” practices.

### 3.1 Current diagnostic pathway in their region

A fast-track diagnostic pathway existed for patients with suspected lung cancer for 33/60 (55 %) of GPs. 8/60 (13 %) of GPs did not have this pathway in their region and 19/60 (32 %) were unsure whether a pathway existed.

**Fig. 1:**
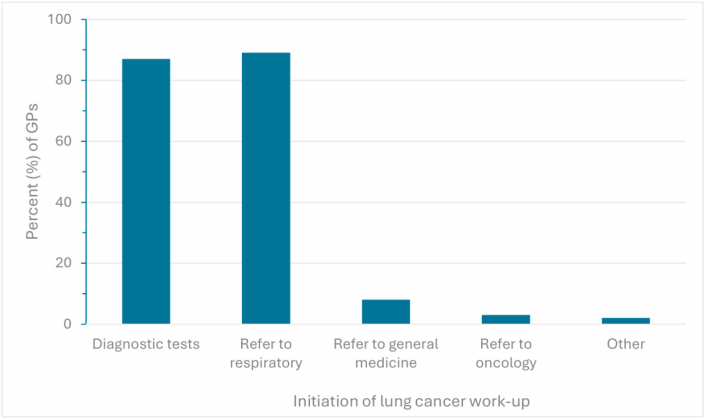
**Lung cancer work-up** General practitioners (GPs) reporting how they initiate work-up of lung cancer. GP responses are shown as percentages.

**Fig. 2:**
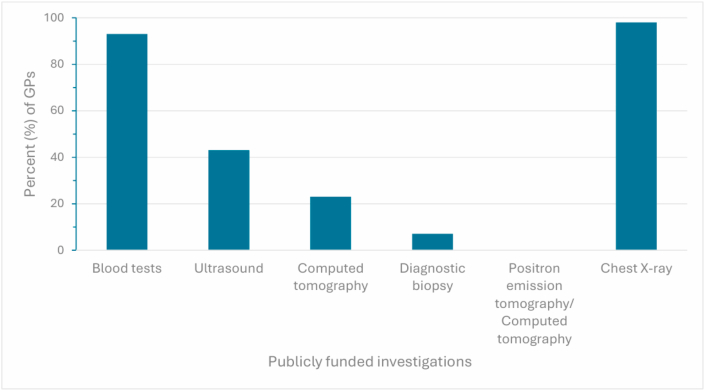
**Tests for lung cancer** Investigations initiated by general practitioners (GPs) for patients with suspected advanced lung cancer. GP responses are shown as percentages.

GPs initiated work-up of lung cancer by ordering diagnostic tests for 53/61 (87 %) of cases and then referring to specialist care (Fig. 1). 60/61 (98 %) of GPs had access to publicly funded chest X-rays and 57/61 (93 %) to diagnostic blood tests. Only 4/61 (7 %) of GPs had access to diagnostic biopsy (Fig. 2).

**Fig. 3:**
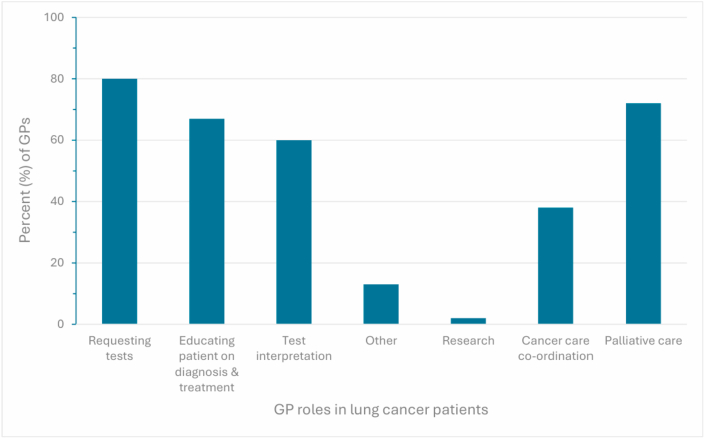
**Roles** The roles of the general practitioner (GP) in managing patients with lung cancer. GP responses are shown as percentages.

The major reported roles of a GP comprised of 49/61 (80 %) requesting tests, 37/61 (61 %) interpreting tests, and 41/61 (67 %) educating the patient around diagnosis and treatment (Fig. 3). Additional obstacles expressed of the current diagnostic pathway included long wait times for GP and/or specialist appointments, wait for CT imaging, as well as limited access to publicly funded CT scans in general practice.

### 3.2 Knowledge about molecular testing, liquid biopsies, and treatment for lung cancer

We briefly surveyed GPs of their familiarity of different modalities of lung cancer treatment. Most GPs were familiar, very familiar or extremely familiar with chemotherapy 51/60 (85 %), surgery 49/61 (81 %) and radiation 52/60 (87 %). Whereas only 24/61 (40 %) and 35/61 (58 %) were similarly confident with molecularly targeted therapy and immunotherapy respectively (Table 1).

**Table 1:** Familiarity with treatments.

	Not familiar at all	Vaguely familiar	Familiar	Very familiar	Extremely familiar	N (total)
Molecularly targeted therapy	25 (41 %)	12 (20 %)	3 (5 %)	9 (15 %)	12 (20 %)	61
Immunotherapy	8 (13 %)	18 (30 %)	9 (15 %)	20 (33 %)	6 (10 %)	61
Chemotherapy	0 (0 %)	9 (15 %)	23 (38 %)	18 (30 %)	10 (17 %)	60
Radiation	0 (0 %)	8 (13 %)	21 (35 %)	18 (30 %)	13 (22 %)	60
Surgery	2 (3 %)	10 (16 %)	17 (28 %)	17 (28 %)	15 (25 %)	61

General practitioners (GPs) rating their experience with lung cancer treatments between “Not familiar at all” and “Extremely familiar”. Results are presented as numbers out of the total response count of N for each treatment and as a percentage (n/N%).

When asked about molecular testing for lung cancer, 51/58 (88 %) of GPs were not aware of molecular testing and 53/58 (91 %) of GPs were not aware of ctDNA liquid biopsy testing for lung cancer. 1/46 (2 %) of GPs had previously used ctDNA liquid biopsy testing. When asked about concerns regarding the incorporation of liquid biopsy into the diagnostic process, some GPs reported concerns regarding subsequent access to secondary care and establishing a culturally appropriate pathway particularly for Māori.

Given the low chance of detecting a germline variant with comprehensive genomic profiling panels, we also asked GPs about their confidence with pre-test genetic counselling. 46/58 (80 %) were not comfortable with pre-test genetic counselling for detection of inherited conditions associated with ctDNA liquid biopsies. Although, 41/58 (71 %) of GPs declared awareness of the genetics referral pathway in New Zealand.

**Fig. 4:**
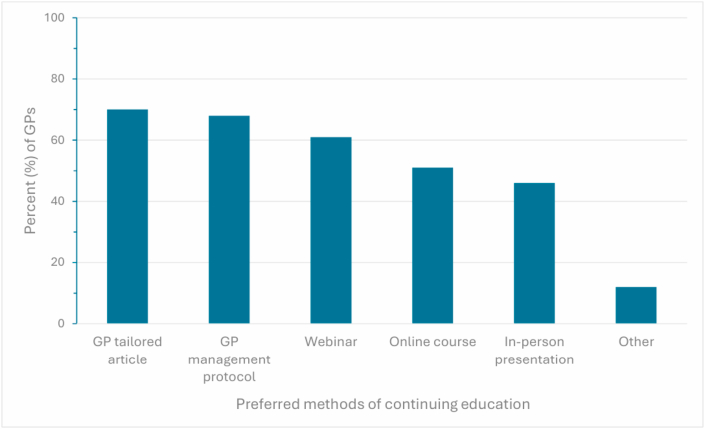
**Continuing education** General practitioners (GPs) preferencing different methods of continued education about liquid biopsy and molecular testing. GP responses are shown as percentages.

This survey aimed to investigate GPs’ attitudes towards initiating plasma based ctDNA as part of the diagnostic pathway for lung cancer. 25/58 (43 %) of GPs initially indicated that they should be able to request ctDNA liquid biopsy testing. However, given the option of adequate training and funding, 42/58 (72 %) of GPs felt comfortable with ordering ctDNA liquid biopsies and explaining the results. The most preferred types of training were 40/57 (70 %) GP-tailored articles, 39/57 (69 %) GP management protocols, 35/57 (61 %) webinars, 29/57 (51 %) online courses, and 26/57 (46 %) in-person presentations as shown in Fig. 4.

## 4 Discussion

Technologies such as liquid biopsy to hasten treatment selection could potentially improve the care of patients with advanced lung cancer. Consistently, liquid biopsy results in faster time to treatment and improved rates of detection when compared to tissue testing alone. There is also potential to reduce inequities by making the diagnostic process accessible in the community, more available in rural areas, or in areas with significant wait times to access secondary care for investigations. We sought to describe the current understanding and perceptions of GPs about the role of liquid biopsies. This is particularly relevant in the NZ model where patients are referred on from GPs to secondary specialist care for complex imaging or diagnostic procedures such as bronchoscopy or percutaneous biopsies.

In our survey we report that most GPs are responsible for initiating the diagnostic process for patients with suspected lung cancer and have access to publicly funded chest X-rays and blood tests. This presents as an opportunity to initiate plasma-based testing that could potentially hasten the diagnostic process. Given that only 33/60 (55 %) of GPs had a fast-track diagnostic pathway in their region, liquid biopsy could be used to supplement the existing pathway. It would be important to assess the areas that do not have a diagnostic pathway more closely to see whether technologies such as liquid biopsy could be utilised in those areas.

Implementation of liquid biopsy does not come without barriers. Of the GPs who responded to the survey, 51/58 (88 %) did not know about molecular testing for lung cancer and only 1/46 (2 %) had previously utilised ctDNA testing. Currently the invasive procedures of bronchoscopy and percutaneous biopsies are mostly limited to secondary care and are initiated by general physicians, respiratory physicians and interventional radiologists. Then the result is usually fed back to the patient by the requesting clinician. We also found a need to improve education on the management of advanced lung cancer for GPs. Patients with lung cancer are often elderly, with multiple medical comorbidities that would benefit from shared care with GPs as well as their oncology teams. Patients are increasingly measured to have extended survival treated with contemporary targeted therapies and immunotherapies, with which GPs reported less familiarity.

Provided there was sufficient education, 42/58 (72 %) of responding GPs felt comfortable with incorporating liquid biopsies into the other tests they initiate in patients with suspected lung cancer. However, currently in NZ there are no government reimbursed liquid biopsy tests. There are several commercially available tests such as the single polymerase chain reaction tests for *EGFR* or more comprehensive genomic profiling but these are limited by out-of-pocket expense for the patient. Moreover, there is no formal molecular tumour board to discuss complex genomic findings and no specific access to genetics counselling for incidental germline findings from testing of tumour.

Some respondents highlighted the importance of having a pathway to access secondary and oncological care to assist with the interpretation and the treatment based on the results. Importantly the implementation of the testing needs to be developed in a culturally appropriate way for our indigenous population. Potentially this technology could reach groups who are currently not engaged with the standard healthcare pathways.

Suggestions to support the adoption of liquid biopsy in general practice include the following. First, development of GP-specific resources, including management protocols and tailored articles which could bridge current knowledge gaps. Second, ensuring public funding for ctDNA tests is critical to avoid exacerbating health inequities. Third, integrating ctDNA testing into existing diagnostic pathways, particularly in regions without fast-track access is essential. Centralised testing with GPs potentially supported by a national molecular tumour board would offer diagnostic accuracy. Fourthly, more extensive consultation is required to ensure the testing method is culturally appropriate for Indigenous groups.

## 5 Conclusion

There is great interest to improve the diagnostic process for lung cancer, especially with the use of emerging technologies such as liquid biopsy. It is important to utilise these technologies in an equitable manner so to improve inequities rather than worsening them [18]. Part of the challenge of implementation of liquid biopsies include improving access, funding and providing training for clinicians in primary care who can initiate these diagnostic tests.

## Declaration of competing interests

The authors declare there are no competing interests.
