# Supplementary material for: General practitioners’ attitudes towards liquid biopsy as a diagnostic test for advanced lung cancer
Source: J Liq Biopsy. 2025 Oct 13;10:100330. doi: 10.1016/j.jlb.2025.100330 (PMC12799498; doi:10.1016/j.jlb.2025.100330)
Supplement: Multimedia component 1 [file mmc1.pdf]

New Zealand Aotearoa GP lung cancer diagnosis and liquid biopsy survey

Clinical Practice Demographics

1) In which region do you practice?

- ◯ Northland
- ◯ Auckland
- ◯ Waikato
- ◯ Lakes District
- ◯ Bay of Plenty
- ◯ Gisborne
- ◯ Hawke's Bay
- ◯ Taranaki
- ◯ Manawatu-Whanganui
- ◯ Wellington
- ◯ Tasman
- ◯ Nelson
- ◯ Marlborough
- ◯ West Coast
- ◯ Canterbury
- ◯ Otago
- ◯ Southland

2) In which setting is your practice?

- ◯ Urban
- ◯ Rural

3) Is your practice a Very Low Cost Access (VLCA) practice?

- ◯ Yes
- ◯ No

Patient Data

4) In the last 12 months, please estimate the number of patients you managed with lung cancer (type in number) __________________________________

5) In the last 12 months, please estimate the number of patients you managed with incurable locally-advanced or metastatic lung cancer (type in number) __________________________________

6) In the last 12 months, for how many patients were you the clinician to initiate diagnostic process for patients with suspected advanced lung cancer? (type in number) __________________________________

Current Diagnosis and Treatment of Lung Cancer

7) What are your roles in managing patients with advanced lung cancer? (tick all that apply)
- ☐ Requesting tests
- ☐ Test interpretation
- ☐ Education of patient about diagnosis and treatment
- ☐ Cancer Care coordination
- ☐ Palliative care
- ☐ Research
- ☐ Other

8) If your answer to the previous question (7) includes the response "Other" please expand on this further. If this does not apply then please continue onto the next question.

____

9) In your region, is there a fast track diagnostic pathway for patients with suspected lung cancer?
- ○ Yes
- ○ No
- ○ Unsure

10) In your service, how do you initiate the work up for a patient with suspected lung cancer? (tick all that apply)
- ☐ Diagnostic tests
- ☐ Refer to respiratory
- ☐ Refer to general medicine
- ☐ Refer to oncology
- ☐ Other

11) If your answer to the previous question (10) includes the response "Other" please specify and/or expand on this further. If this does not apply then please continue onto the next question.

____

12) What publicly funded tests can you request?
- ☐ CXR (Chest X-Ray)
- ☐ US (Ultrasound)
- ☐ CT (Computerized Tomography Scan)
- ☐ PET/CT (Positron Emission Tomography/Computerized Tomography)
- ☐ Diagnostic biopsy
- ☐ Blood tests

13) Are you familiar with different types of treatment for lung cancer? (Please rank the below treatments between 1-5, where 1 = Extremely familiar to 5 = Not familiar at all)

	1	2	3	4	5
Surgery	○	○	○	○	○
Radiation	○	○	○	○	○
Chemotherapy	○	○	○	○	○
Immunotherapy	○	○	○	○	○
Molecularly targeted therapy	○	○	○	○	○

14) What do you think are the main barriers in the diagnostic process for advanced lung cancer currently?

____

Blood Based Testing

Some lung cancers have a cancer-related (actionable) mutation that can be inhibited by oral targeted therapies. The identification of this actionable mutation relies on a percutaneous biopsy and subsequent molecular testing. Recently, blood-based testing for circulating tumour DNA (ctDNA) to identify these actionable mutations are being refined. Potentially these tests can increase the chance of detecting a mutation by approximately 15% compared to percutaneous biopsy alone. The following questions are about this blood based testing.

15) Are you aware of molecular testing for lung cancer? (if Yes, go to Q16; If No, go to Q17)
- ◯ Yes
- ◯ No

16) Have you requested any molecular testing for patients with advanced lung cancer e.g. Epidermal Growth Factor Receptor (EGFR) or Anaplastic Lymphoma Kinase testing?
- ◯ Yes
- ◯ No

17) Are you aware of circulating tumour DNA (ctDNA) liquid biopsies for cancer diagnosis and treatment? (if Yes, go to Q18; if No, go to Q19)
- ◯ Yes
- ◯ No

18) Have you used blood-based ctDNA tests before?
- ◯ Yes
- ◯ No

19) Who should be able to request these blood-based ctDNA tests? (tick all apply)
- ☐ GP
- ☐ Other primary health care providers e.g. Nurse practitioner
- ☐ Respiratory
- ☐ Oncology
- ☐ Other

20) If your answer to the previous question (19) includes the response "Other" please specify this further in the text box provided. If this does not apply then please continue onto the next question.

____

21) Some comprehensive genomic testing or ctDNA liquid biopsies can detect inherited conditions that can increase the chance of cancer. Do you feel comfortable with pre-test genetic counselling of these conditions?
- ◯ Yes
- ◯ No

22) Are you aware of the genetics referral pathway in NZ?
- ◯ Yes
- ◯ No

23) If there was adequate funding and training, would you feel comfortable requesting blood-based ctDNA tests and explaining results?
- ◯ Yes
- ◯ No

24) How much time would be adequate to explain blood based ctDNA test result?
- ◯ 15 min
- ◯ 30 min
- ◯ 45 min
- ◯ 60 min

25) What is your most preferred method(s) of continued medical education about ctDNA? (tick all that apply)
- ☐ Webinar
- ☐ In-person presentation
- ☐ Online course
- ☐ Health pathways
- ☐ BPAC article
- ☐ Other

26) If your answer to the previous question (25) includes the response "Other" or you would like to expand on your response, please specify further in the text box provided. __________________________________
